# Impact of the external school food environment on the associations of internal school food environment with high schoolers’ diet and BMI

**DOI:** 10.1017/S1368980022000994

**Published:** 2022-11

**Authors:** Grace M Betts, Carolina Schwedhelm, Leah M Lipsky, Denise L Haynie, Tonja R Nansel

**Affiliations:** 1 Social and Behavioral Sciences Branch, Division of Intramural Population Health Research, Eunice Kennedy Shriver National Institute of Child Health and Human Development, National Institutes of Health, Bethesda, MD 20817, USA; 2 Max-Delbrueck-Center for Molecular Medicine in the Helmholtz Association (MDC), Molecular Epidemiology Research Group, Berlin, Germany

**Keywords:** School food availability, Food environment, Food outlets, Adolescents, BMI, Food intake

## Abstract

**Objective:**

To examine associations of school food availability with student intake frequency and BMI, and whether the number of neighbourhood food outlets modifies these associations.

**Design:**

Baseline assessment of a nationally representative cohort study of US 10th graders. Students reported intake frequency of fruits and vegetables (FV), snacks and soda. BMI was calculated from measured height and weight. Administrators of seventy-two high schools reported the frequency of school availability of FV, snacks and soda. The number of food outlets within 1 km and 5 km were linked with geocoded school addresses. Data were analysed using adjusted linear and logistic mixed models with multiple imputation for missing data.

**Setting:**

US 2009–2010.

**Participants:**

Totally, 2263 US 10th graders from the Next Generation Health Study (NEXT).

**Results:**

Greater school FV availability was positively associated with student FV intake. Food outlets within 5 km of schools (but not 1 km) attenuated the association of school FV availability with student intake; this was no longer significant at schools with > 58 food outlets within 5 km. School food availability was not associated with student BMI or student snack or soda intake.

**Conclusions:**

School food availability was associated with student intake of FV, but not with snacks, soda or BMI. Attenuation of the observed associations by the school neighbourhood food environment indicates a need to find ways to support healthy student eating behaviours in neighbourhoods with higher food outlet density.

One-fifth of US children and adolescents aged 12–19 are obese^(1)^ and few meet national dietary guidelines for a healthy diet^(2)^. Obesity in adolescence is associated with increased risk of adult obesity^(3)^ and related adverse health outcomes including type 2 diabetes, CVD and premature death^(4)^. Strategies targeting behavioural change to prevent obesity in childhood and adolescence often fail in food environments that promote high energy intake and sedentary behaviour^(5)^. The internal school food environment (e.g. school lunches and competitive foods available within schools) has frequently been targeted for improving youth diet and weight outcomes. Youths consume over one-third of their daily food in school^(6)^, and students who eat both breakfast and lunch at school consume close to 50 % of their total intake at school^(7)^. Additionally, the external school food environment (i.e. foods available in the school’s surrounding neighbourhood) becomes increasingly relevant for high schoolers, who have increased autonomy over food decisions and may be less constrained to the internal school food environment than middle schoolers^(8)^.

Policies impacting the provision, restriction and nutrient content of school lunches and competitive foods and beverages (i.e. foods and beverages offered outside of school meal programmes) in middle and high schools are associated with lower intake of restricted foods and beverages (e.g. lower salty snack and soda intake)^(9–16)^, although evidence suggests that students compensate by acquiring restricted foods from alternative sources^(17)^ or consuming greater amounts of unrestricted foods of lower nutritional value while in school^(10)^. Additionally, school policies restricting competitive foods sometimes allow schools to replace restricted foods with similar foods lacking nutritional value (e.g. regular potato chips *v*. baked) and fail to require healthier alternatives (e.g. vegetables and whole grains)^(13)^. The effects of these school nutrition policies on BMI (kg/m^2^) have been mixed. Stricter policies have been associated with improved weight status among 10–19-year-olds^(9,18–20)^; in contrast, associations of stricter nutrition policies with greater odds of overweight and obesity were observed in elementary and middle school students^(21,22)^, while null associations have been observed in high school students^(21,23)^ and middle school students^(24)^.

Evidence suggests the internal school food environment may have a smaller impact on diets and weight outcomes of high school students than of those in younger grades^(16,23)^ due in part to greater influence of the external school food environment^(25)^. High school students have access to neighbourhood food outlets (e.g. restaurants, grocery stores, convenience stores and gas stations) before and after school and are often permitted to leave campus during lunch^(25)^. Additionally, data indicate that businesses, including fast-food restaurants, are more frequently located within close proximity to high schools than elementary or middle schools^(25)^, and that closer proximity and higher density of food outlets (primarily fast-food restaurants and convenience stores) in school and home neighbourhoods are associated with less healthy food intake^(26–28)^ and higher BMI^(26,28)^ in middle and high school students. However, other studies have found null associations of neighbourhood food environments with youth diet and weight outcomes^(26,27,29)^. Furthermore, few studies have examined associations of the external school food environment with BMI in high school students^(28–30)^, and none have concurrently examined the internal and external food environment. As such, it is unknown whether the external school food environment modifies the relationship of the internal school food environment with student diet and weight outcomes. Thus, the purpose of this study is to investigate whether the neighbourhood food environment modifies the association of school food availability with student dietary intake and BMI. We hypothesised that more food outlets in the external school food environment would weaken associations of the internal school food environment with student intake and BMI.

## Methods

### Study design

Data come from the baseline assessment of the Next Generation Health Study (NEXT), a nationally representative cohort study of 10th graders enrolled during the 2009–2010 school year and assessed annually for 7 years (*n* 2783). The primary sampling units were school districts stratified across the nine US Census divisions; 81 of 137 schools (public and private) randomly selected agreed to participate, and classrooms from among core subject matter classes within these schools were randomly selected for inclusion. Schools were located in twenty-two states, with at least two states from each census division included. Additional study details are available elsewhere^(31)^. Of the eighty-one schools included in NEXT, seventy-two reported on school food availability (*n* 2263) and therefore were included in this analysis. Schools with large percentages of African-American students were oversampled to provide reliable estimates for this subgroup; a sufficient number of Hispanic students were obtained to provide reliable subgroup estimates without oversampling. While neighbourhood characteristics of schools reporting on food availability were not different from the NEXT full sample, the proportions of White and Black students were slightly higher and lower, respectively, in the analytic sample than in the NEXT full sample; there were no other demographic differences (Supplementary Table 1). Students completed self-administered surveys annually and school administrators completed a self-administered survey at baseline (wave 1). All data used in this paper come from wave 1, except for wave 2 intake frequency of sweet and salty snacks (data not available at baseline). Parents provided written informed consent for their child and students provided assent (if < 18 years of age) and consent (if ≥ 18 years of age). Study protocol was approved by the Institutional Review Board of the Eunice Kennedy Shriver National Institute of Child Health and Human Development.

### Measures

#### Anthropometric and demographic data

Students self-reported race/ethnicity (categorised as White, Black, Hispanic and other) and gender (male/female); age was calculated based on birthdate reported by parents during the consent process. Family affluence was calculated using the Family Affluence Scale, a validated indicator of family wealth based on participant responses to questions regarding their household car and computer ownership, family vacation, and bedroom sharing^(32)^. A categorical, composite Family Affluence Scale score was used for analysis, with scores 0–2 indicating low, 3–5 middle and 6–9 high affluence^(33)^. Parent education was reported by the parent completing the consent process and was categorised as high school/GED or less, some college/technical school/associate’s degree, and bachelor’s degree or higher. In two-parent families, the higher parent education was used. Height and weight were measured by trained study staff and were recorded to the nearest 0·1 cm and 0·1 kg, respectively. Measured height and weight were used to calculate BMI. Where BMI was missing at wave 1, BMI at wave 2 was used (from *n* 118 missing at wave 1, *n* 29 were available at wave 2). BMI was previously shown to be fairly consistent across waves^(34)^.

#### School food availability

School administrators completed a survey, including the question, ‘Can students purchase any of the following items (fruits, vegetables, 100 % fruit juice, chocolate candy, other candy, regular salty snacks, low-fat salty snacks, regular sweet snacks, low-fat sweet snacks, soda) from school vending machines or at the school store, cafeteria, or snack bar?’ with response options of no, yes-some days and yes-daily. Responses to availability of fruits, vegetables and 100 % fruit juice were each scored as 0 = no days, 1 = some days and 2 = daily. Overall school fruit and vegetable (FV) availability was calculated by summing the frequency of the availability of each of these items and ranged from 0 (no fruits, vegetables or 100 % fruit juice offered any day) to 6 (fruits, vegetables and 100 % fruit juice offered daily). Responses to snack items were categorised as chocolate and candy (chocolate + other candy), salty snacks (low-fat + regular salty snacks) and sweet snacks (low-fat + regular sweet snacks), each of which was scored according to the most frequently available snack in each category as 0 = no days, 1 = some days and 2 = daily. Overall school snack availability was calculated by summing the frequency of the availability of each snack category and ranged from 0 (no snacks offered any day) to 6 (snacks from all categories offered daily). Soda availability was based on the original three response options for this item: 0 = no days, 1 = some days and 2 = daily.

#### External school food environment (food outlets)

Food outlet counts within 1 km and 5 km were obtained from business location data provided by Dun & Bradstreet (www.dnb.com) and linked with the school geocoded addresses. These distances correspond to a 10–15 min walk (1 km) and a 5–10 min drive (5 km), respectively, representing distances that may be reasonably reached by students before, during or after school^(35,36)^. Fast-food outlets (chain and independent), full-service restaurants, convenience stores and grocery stores/supermarkets were summed to calculate the total number of food outlets (hereinafter, all food outlets). Analyses by food outlet category were examined individually for these four outlet types.

#### Other school neighbourhood measures

Population density and poverty rate by census block were obtained from the 2010 US Census and American Community Survey^(37)^. Census blocks are delineated by the US Census Bureau and are defined as neighbourhood areas bounded by visible and non-visible features, such as roads, streams and county limits and are the basis for all tabulated data^(38)^. Geographic information system data were used to calculate land use mix scores ranging from 0 to 1 that describe the diversity of land use by ZIP code tabulation area of schools. Scores of 0 and 1 represent the most homogeneous and most diverse land use, respectively^(39)^.

#### Student food group intake frequency

Student food group intake frequency was assessed using questions based on the Youth Risk Behavior Surveillance System^(40)^ and the multinational Health Behaviour in School-Aged Children study^(41)^. Participants were asked, ‘During the past 7 d, how many times did you eat or drink (sweet and salty snacks (assessed at wave 2), soda, and fruits and vegetables)?’ Responses ranged from never to four or more times per d. FV intake frequency was calculated by summing responses to 100 % fruit juices, fruit, green salad, carrots and other vegetables. Responses for all food groups were converted to number of times per d. Due to low intake frequency of snacks and soda resulting in a right-skewed distribution, variables for intake of these food groups were dichotomised as less than one time per d and greater or equal to one time per d.

### Analysis

Multiple imputation by chained equations was used to impute missing values under the assumption of missing at random. Missing values were present for gender (*n* 4, 0·2 %), age (*n* 15, 0·7 %), BMI (*n* 89, 3·9 %), race/ethnicity (*n* 8, 0·4 %), parental education (*n* 139, 6·1 %), soda intake (*n* 29, 1·3 %) and snack intake (*n* 309, 13·7 %). Using R package ‘mice’, fifty imputed datasets were generated and imputed values for missing variables were derived from their estimated distribution conditional on other variables. Analyses were repeated per imputed dataset and estimates were combined by Rubin’s rule^(42)^.

School and student characteristics were summarised using frequency and percent, and mean and standard error for categorical and continuous values, respectively. Due to the skewed distribution, the median and interquartile range of food outlets were reported. Separate models examined the total number of food outlets within 1 km and 5 km of schools to assess whether the school neighbourhood food environment modified the association of school food and drink availability with student BMI and food group intake frequency. For models indicating a statistically significant interaction, regions of significance and interaction plots further examined the interaction, and additional analyses examined food outlet categories separately (fast-food outlets, full-service restaurants, convenience stores and grocery stores/supermarkets). Linear mixed models were used to examine the association of school food and drink availability (FV, snacks and soda) with student BMI and to examine the association of school FV availability with student FV intake. Logistic mixed models were used to examine associations of school snack and soda availability with student snack and soda intake frequency. All models included sample weight to account for survey design and random intercepts for schools to account for clustering within schools. All models adjusted for student race/ethnicity, family affluence and highest parent education, as well as school neighbourhood land use mix, population density and poverty rate. Adjustment variables were selected based on literature; bivariate correlations were examined to avoid multicollinearity and bivariate associations were examined for dependent variables and demographic characteristics (see online supplementary material, Supplemental Table 2). The Johnson–Neyman technique was applied using R package ‘interactions’ to probe significant interactions and determine regions of significance^(43)^, identifying for which values of the moderator (number of food outlets) significance held. However, a limitation of this approach is that statistical significance at higher levels of the moderator may occur as an artefact of the analysis. All analyses were performed in R (version 4.0.1, R Foundation for Statistical Computing) and interaction plots were generated with SAS (version 9.4, Enterprise Guide 7.1, SAS Institute Inc.).

## Results

Of the eighty-one schools included in NEXT, seventy-two reported on school food availability (*n* 2263 students). Participants were on average 16 years old (ranging from 14 to 20 years; 98 % were between 15 and 17 years), approximately half female and approximately 40 % racial/ethnic minority. Family affluence and parent education were well distributed across the three categories of each variable (Table 1).

**Table 1:** Individual and school neighbourhood characteristics

	Sample and school characteristics		
	Students (*n* 2263)		
	Mean	%	se
Age (years)	16·26		0·01
Gender (% female)		54·25	1·66
BMI (measured)	24·78		0·13
Race/ethnicity (%)			
White		59·23	1·62
Black		16·07	1·14
Hispanic		19·61	1·30
Other		5·09	0·77
Parent education (%)			
High school, GED, or less		33·69	1·58
Some college/tech school/associates		39·48	1·67
Bachelor’s degree or higher		26·83	1·50
Family affluence (%)			
Low affluence		24·04	1·36
Moderate affluence		48·76	1·66
High affluence		27·20	1·53
FV intake (times/d)	3·90		0·08
Snack intake, times/d (%)			
< 1		54·35	1·73
≥ 1		45·65	1·73
Soda intake, times/d (%)			
< 1		61·11	1·62
≥ 1		38·89	1·62
Schools (*n* 72)			
Neighbourhood land use mix*	0·45		0·04
Neighbourhood population density (per square mile)	7213·08		2335·58
Neighbourhood poverty rate†	35·07		2·51
School FV availability score (%)	4·07		0·24
0 (no days)		6·94	3·02
1–2		26·39	5·23
3–5		20·84	4·82
6 (all components daily)		45·83	5·91
School snack availability score (%)	3·61		0·24
0 (no days)		11·11	3·73
1–2		20·84	4·82
3–5		37·50	5·75
6 (all components daily)		30·56	5·47
School soda availability score (%)	1·06		0·11
0 (no days)		41·67	5·85
1 (some days)		11·11	3·73
2 (daily)		47·22	5·92

	Median	IQR	Range
Neighbourhood food outlets within 1 km, median (IQR) and range			
All food outlets	2	0–12	0–335
Fast-food restaurants	0	0–2	0–20
Full-service restaurants	1	0–4·5	0–234
Convenience stores	0	0–1	0–16
Supermarkets/grocery stores	1	0–4	0–92
Neighbourhood food outlets within 5 km, median (IQR) and range			
All food outlets	13·5	5–242·5	0–2828
Fast-food restaurants	4·5	0·5–32·5	0–154
Full-service restaurants	6·5	1–112	0–1831
Convenience stores	2	0–13·5	0–129
Supermarkets/grocery stores	4	1–66·5	0–974

FV, fruit and vegetable; IQR, interquartile range.

Values are mean or % (se), or median (IQR) and range.

Weighted means and percentages are reported for all student-level variables. Snack intake measured at wave 2; all others measured at wave 1 (baseline).

* A score of 0–1 measured the diversity of land use in schools’ ZIP code tabulation area with 0 representing the most homogeneous land use and 1 representing the most diverse land use.

† Percent of the population with income of less than 185 % of the Federal Poverty Level.

Approximately half of schools offered fruits, 100 % fruit juice and vegetables daily, nearly one-third offered snacks from each category (chocolate and candy, salty snacks, sweet snacks) daily and nearly half offered soda daily (Table 1). On average, students reported eating FV approximately four times/d, and a majority reported drinking soda or eating snacks < 1 time/d. Schools had a median of 2 (interquartile range: 0–12) food outlets within 1 km and almost 14 (IQR: 5–242·5) within 5 km. Schools with the highest numbers of food outlets within 5 km (> 1000 food outlets) were deemed plausible; these schools (*n* 4) were all in highly urbanised areas of New York, with land use mix scores above the median. Analyses excluding these schools did not change the findings (results not shown).

### School food availability and student intake: all school neighbourhood food outlets

**Fig. 1:**
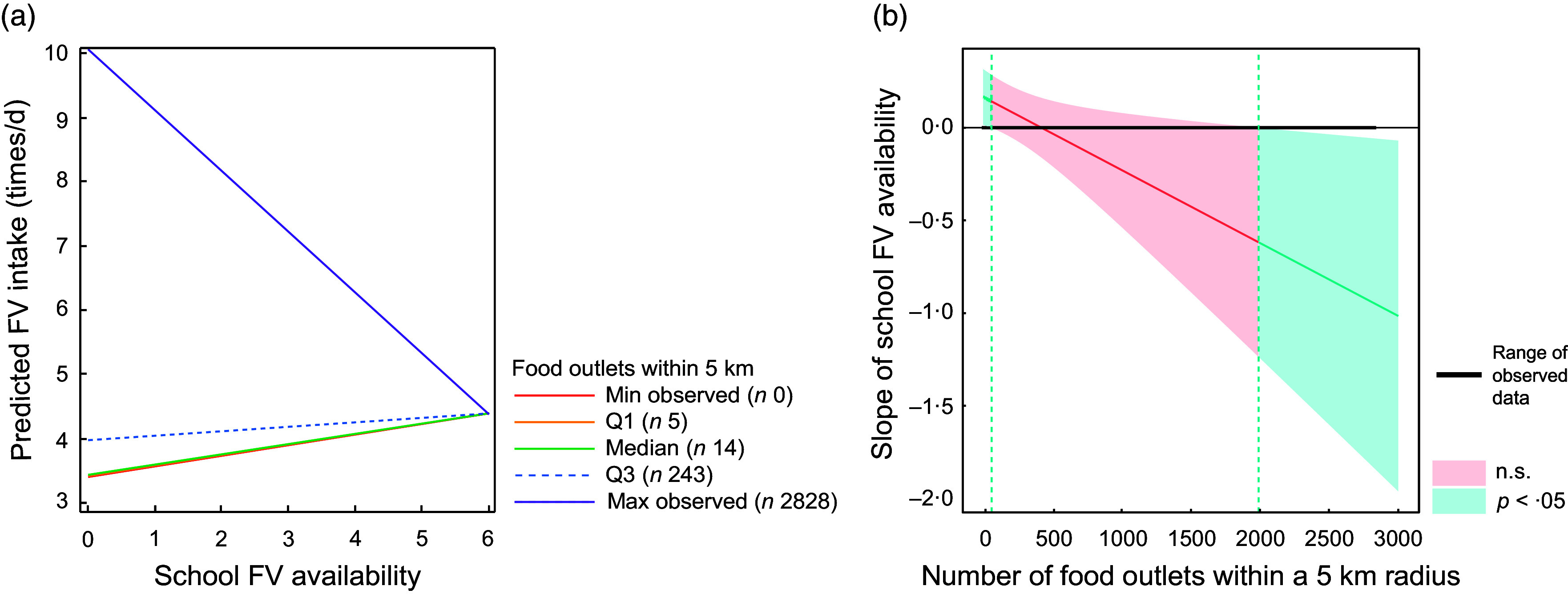
(a) Interaction plot showing simple slopes of the regression of school FV availability on student FV intake at different levels of all food outlets within a 5-km radius of schools. Solid lines indicate that the slopes are within the regions of significance (*P* < 0·05) presented in Fig. 1(b), while dashed lines indicate the slopes are outside of the regions of significance (*P* ≥ 0·05) presented in Fig. 1(b). (b) Johnson–Neyman regions of significance and confidence bands (95 % CI) for the conditional relation between school FV availability and student FV intake as a function of all food outlets within 5 km of schools. Blue shaded areas reflect regions of significance ((0, 58·9) and (> 1990·2)) and the bold horizontal line indicates the range of observed food outlets in the sample data (0–2828). When the number of food outlets is between 0 and 58 and 1991 and higher, the slope of school FV availability is *P* < 0·05. FV, fruit and vegetable

In models investigating all school neighbourhood food outlets within a 1-km radius, school FV availability was positively associated with student FV intake (*β*: 0·15; 95 % CI: 0·002, 0·29) meaning that for every one unit increase in school FV availability students ate FV 0·15 more times per d. The number of food outlets within 1 km was not associated with student FV intake, and there was no interaction of food outlets with school FV availability (Table 2). In models investigating food outlets within a 5-km radius, school FV availability and food outlets were both positively associated with student FV intake (*β*: 0·17; 95 % CI: 0·02, 0·31), and food outlets attenuated the positive association of school FV availability with student FV intake (Fig. 1(a)). Regions of significance showed that the positive association of school FV availability with student FV intake occurred at schools with less than 59 and with over 1990 food outlets within 5 km (Fig. 1(b)). In schools with 59–1990 food outlets within 5 km, school FV availability was not associated with intake. School availability of snacks and soda was not associated with intake.

**Table 2:** Model estimates of the associations of school food and drink availability (frequency) and the school neighbourhood food environment (count of total food outlets within 1 km and 5 km), as well as their interaction, with corresponding student intake frequency (times/d) of fruits and vegetables (FV), snacks, and soda

	FV intake (times/d)		Snacks intake (≥ 1 time/d)†		Soda intake (≥ 1 time/d)	
Independent variables	*β*	95 % Cl	OR	95 % CI	OR	95 % CI
Model 1: 1 km school food environment						
School availability‡	0·15	0·002, 0·29*	1·01	0·94, 1·08	1·03	0·87, 1·22
All food outlets	0·02	–0·02, 0·07	1·00	0·99, 1·01	1·00	0·99, 1·01
School availability × All food outlets	−0·004	–0·01, 0·002	1·00	1·00, 1·01	1·00	0·99, 1·01
Model 2: 5 km school food environment						
School availability	0·17	0·02, 0·31*	1·02	0·95, 1·09	1·04	0·88, 1·23
All food outlets	0·002	0·0002, 0·004*	1·00	0·99, 1·01	1·00	0·99, 1·01
School availability × All food outlets	−0·0004	–0·001, –0·0001*	1·00	1·00, 1·00	1·00	1·00, 1·01

Linear (FV intake) and logistic (snack and soda intake) mixed models were used to calculate estimates.

Models adjusted for race/ethnicity (ref = White), parent education (ref = high school or less), family affluence (ref = low affluence), neighbourhood land use mix, neighbourhood population density and neighbourhood poverty rate.

* *P* < 0·05.

† Snack intake measured at wave 2; all others measured at wave 1 (baseline).

‡ School availability of the food group corresponding to the outcome variable (e.g. school fruit and vegetable availability with student fruit and vegetable intake frequency). FV availability score ranges from 0 (no fruits, vegetables or 100 % fruit juice offered any day) to 6 (fruits, vegetables and 100 % fruit juice offered every day); snacks availability score ranges from 0 (no chocolate and candy, salty snacks or sweet snacks offered any day) to 6 (chocolate and candy, salty snacks and sweet snacks offered every day); soda availability score ranges from 0 (no soda offered any day) to 2 (soda offered every day).

### School availability and student intake of fruits and vegetables: food outlet categories

#### Fast-food outlets

Fast-food outlets within 1 km were positively associated with student FV intake (*β*: 0·33; 95 % CI: 0·03, 0·63) (Table 3) but attenuated the positive association between school availability and student intake (see online supplementary material, Supplemental Fig. 1(a)). Regions of significance showed that the positive association of school FV availability with student FV intake was observed at schools with zero and with over ten fast-food outlets (but not 1–10 outlets) within 1 km (see online supplementary material, Supplemental Fig. 1(b)). Similarly, fast-food outlets within a 5-km radius were positively associated with student FV intake (*β*: 0·05; 95 % CI: 0·02, 0·08) (Table 3) and attenuated the positive association of school FV availability with student FV intake (see online supplementary material, Supplemental Fig. 1(c)). Regions of significance showed that this relationship was observed at schools with less than twelve and with over fifty-two fast-food outlets within 5 km, but not at schools with 12–52 fast-food outlets within 5 km (see online supplementary material, Supplemental Fig. 1(d)).

**Table 3:** Model estimates of the associations of school FV availability (frequency) and the school neighbourhood food environment (count of food outlets within 1 km and 5 km) by food outlet categories, as well as their interaction, with student FV intake frequency (times/d)

	Fast-food outlets		Full-service restaurants		Convenience stores		Grocery stores/supermarkets	
	FV intake (times/d)							
Independent variables	*β*	95 % Cl	*β*	95 % Cl	*β*	95 % Cl	*β*	95 % Cl
Model 1: 1 km school food environment								
School FV availability†	0·16	0·01, 0·31*	0·15	0·003, 0·29*	0·14	–0·002, 0·29	0·15	–0·002, 0·29
Food outlets	0·33	0·03, 0·63*	0·06	–0·05, 0·18	−0·12	–0·42, 0·19	0·04	–0·03, 0·11
School FV availability × Food outlets	−0·07	–0·13, –0·01*	−0·01	–0·03, 0·01	−0·02	–0·05, 0·02	−0·01	–0·02, 0·002
Model 2: 5 km school food environment								
School FV availability	0·24	0·08, 0·40*	0·16	0·02, 0·31*	0·16	0·01, 0·31*	0·15	0·002, 0·30*
Food outlets	0·05	0·02, 0·08*	0·01	0·001, 0·01*	0·03	–0·01, 0·06	0·004	–0·001, 0·01
School FV availability × Food outlets	−0·01	–0·01, –0·004*	−0·001	–0·002, –0·0002*	−0·005	–0·01, –0·00001*	−0·001	–0·001, 0·0001

FV, fruit and vegetable.

Linear mixed models were used to calculate estimates.

Models adjusted for race/ethnicity (ref = White), parent education (ref = high school or less), family affluence (ref = low affluence), neighbourhood land use mix, neighbourhood population density and neighbourhood poverty rate.

* *P* < 0·05.

† School availability of the food group corresponding to the outcome variable (e.g. school fruit and vegetable availability with student fruit and vegetable intake frequency). FV availability score ranges from 0 (no fruits, vegetables or 100 % fruit juice offered any day) to 6 (fruits, vegetables and 100 % fruit juice offered every day); snacks availability score ranges from 0 (no chocolate and candy, salty snacks or sweet snacks offered any day) to 6 (chocolate and candy, salty snacks and sweet snacks offered every day); soda availability score ranges from 0 (no soda offered any day) to 2 (soda offered every day).

#### Full-service restaurants

The number of full-service restaurants within 1 km was not associated with student FV intake, and there was no statistically significant interaction between the number of full-service restaurants and school FV availability (Table 3). Full-service restaurants within a 5-km radius were positively associated with student FV intake (*β*: 0·01; 95 % CI: 0·001, 0·01) (Table 3) but attenuated the positive association between school FV availability and student FV intake (see online supplementary material, Supplemental Fig. 2(a)). Regions of significance showed that this relationship was observed at schools with less than 26 and with over 737 full-service restaurants within 5 km, but not at schools with 27–737 full-service restaurants within 5 km (see online supplementary material, Supplemental Fig. 2(b)).

#### Convenience stores

In models investigating convenience stores within a 5-km radius, the association of convenience stores with student FV intake did not reach statistical significance (Table 3), but the number of convenience stores within a 5-km radius attenuated the positive association between school FV availability and student FV intake (see online supplementary material, Supplemental Fig. 3(a)). Regions of significance showed that this relationship was observed at schools with up to three convenience stores within 5 km, but not at schools with over three convenience stores within 5 km (see online supplementary material, Supplemental Fig. 3(b)). Although results were consistent in the 1-km and 5-km models, the associations did not reach statistical significance in the model investigating convenience stores within a 1-km radius.

#### Grocery stores/supermarkets

The number of grocery stores/supermarkets within 1 km and 5 km was not associated with student FV intake, and there was no interaction of grocery stores/supermarkets with school FV availability (Table 3).

#### School food availability and student BMI: all school neighbourhood food outlets

Neither school FV availability, school snack and soda availability, nor the total number of school neighbourhood food outlets was associated with student BMI (Table 4).

**Table 4:** Model estimates of the associations of school food and drink availability (frequency) and the school neighbourhood food environment (count of total food outlets within 1 km and 5 km), as well as their interaction, with student BMI (kg/m^2^)

	School food and drink availability* †					
	FV		Snacks		Soda	
	Student BMI (kg/m^2^)					
Independent variables	*β*	95 % Cl	*β*	95 % Cl	*β*	95 % Cl
Model 1: 1 km school food environment						
School availability	0·14	–0·12, 0·41	−0·08	–0·34, 0·19	0·10	–0·43, 0·63
All food outlets	0·01	–0·07, 0·10	−0·001	–0·03, 0·03	−0·002	–0·03, 0·02
School availability × All food outlets	−0·002	–0·01, 0·01	−0·00001	–0·02, 0·02	−0·01	–0·03, 0·02
Model 2: 5 km school food environment						
School availability	0·17	0·10, 0·44	−0·06	–0·32, 0·21	0·14	–0·39, 0·68
All food outlets	0·002	–0·23, 0·53	0·0002	–0·002, 0·002	−0·0001	–0·002, 0·002
School availability × All food outlets	−0·0003	–0·09, 0·03	−0·0002	–0·001, 0·001	−0·001	–0·003, 0·001

FV, fruit and vegetable.

Linear mixed models were used to calculate estimates.

Models adjusted for race/ethnicity (ref = White), parent education (ref = high school or less), family affluence (ref = low affluence), neighbourhood land use mix, neighbourhood population density and neighbourhood poverty rate.

* *P* < 0·05.

† School availability of the food group corresponding to the outcome variable (e.g. school fruit and vegetable availability with student fruit and vegetable intake frequency). FV availability score ranges from 0 (no fruits, vegetables or 100 % fruit juice offered any day) to 6 (fruits, vegetables and 100 % fruit juice offered every day); snacks availability score ranges from 0 (no chocolate and candy, salty snacks or sweet snacks offered any day) to 6 (chocolate and candy, salty snacks and sweet snacks offered every day); soda availability score ranges from 0 (no soda offered any day) to 2 (soda offered every day). Snack intake measured at wave 2; all others measured at wave 1 (baseline).

## Discussion

In this nationally representative sample of US adolescents, greater school FV availability was positively associated with student FV intake. The number of total food outlets (including full-service restaurants, fast-food outlets, convenience stores, and supermarkets and grocery stores) within a 5-km radius of schools attenuated the association of school FV availability with student intake. The positive association of school FV availability with student FV intake was non-significant when there were more than fifty-eight neighbourhood food outlets within 5 km. Because different foods are available from different food outlets, we further examined this association by food outlet type and found that fast-food outlets (within 1 and 5 km of schools) and full-service restaurants and convenience stores (within 5 km of schools), but not grocery stores/supermarkets attenuated the association of school FV availability with student FV intake. Neither the number of school neighbourhood food outlets nor school availability of snacks and soda was associated with student snack and soda intake, and neither school food availability nor neighbourhood food outlets were associated with student BMI.

No previous studies have assessed whether the external school food environment (i.e. food outlets in school neighbourhoods) moderates the association of the internal school food environment (i.e. availability of foods and beverages within schools) with student dietary intake. Our findings suggest that the school’s external food environment may limit the potential impact of the internal school food environment on student outcomes; this was the case for food outlets within 5 km but not 1 km of schools, which could be due to limited observations with a substantial number of food outlets within 1 km (50 % of schools had two or fewer food outlets within 1 km). Furthermore, the significant interactions observed at the upper range of food outlets may be due to constraints of a linear model (i.e. an interaction will eventually lead to a predictor slope of 0 and crossover to a reversed association), limiting interpretability. Evidence regarding the association of proximity of food outlets to schools with student FV intake has been mixed^(27,28,44)^; our finding of a positive association raises the question of whether the external school food environment could be leveraged to promote healthful choices targeted to students. A possible explanation for the finding that grocery stores/supermarkets did not modify the association of school FV availability with student FV intake may be better access to small stores around schools than to larger grocery stores^(44)^. The finding that school FV availability was positively associated with student FV intake at schools with fewer than fifty-nine food outlets within 5 km is consistent with previous studies that found school policies targeting the provision of healthy foods were associated with greater intake of those foods^(14,16)^. In the present study, neither the internal nor external school food environment was associated with student snack or soda intake. Although this contradicts findings from previous studies reporting significant associations of policies limiting the sale of unhealthy foods and drinks in schools with lower intake of those items^(10–12,14,15)^, many of these studies focused on in-school consumption^(10,14,15)^. This suggests that consumption of these foods outside school may counteract the impact of in-school restrictions on students’ overall diet^(10,14,17)^. Similarly, we did not find associations of the internal or external school food environment with student BMI. Although stricter school nutrition policies have been associated with improved weight outcomes^(9,18–20)^, our results are consistent with studies reporting null associations^(21,23)^. However, the positive association of school FV availability with student FV intake only in the presence of low numbers of surrounding food outlets is a novel finding that suggests that the external school food environment modifies the relationship of the internal school food environment with adolescent food intake in some contexts. Targeted strategies may be needed to increase student FV intake in schools with higher neighbourhood food outlet density.

These findings should be interpreted in consideration of the strengths and limitations of the study design. Strengths include the nationally representative sample, which supports the generalisability of the findings. Additionally, the large sample size, use of directly measured BMI, and assessment and control for multiple neighbourhood covariates strengthen the internal validity of the findings. However, limitations to internal validity include the lack of detailed information on food outlet data (i.e. criteria used to define food outlets as fast-food outlets, full-service restaurants, convenience stores, and supermarket and grocery stores) and the observational study design and cross-sectional analysis, precluding determination of directionality and therefore inferences regarding causality. Despite the longitudinal design of the study, data on the internal and external school food environment were only available at baseline. Although student demand may influence the internal and external school food environment, it is unlikely there would be sufficient change in school policies or the external environment to implement longitudinal analyses using the current study design. Finally, our cross-sectional analyses included some data from wave 2, which may have slightly attenuated the associations.

In conclusion, these findings based on a national sample of US 10th graders indicate that the school neighbourhood food environment may modify the relationship of the school food environment with student eating behaviours. Additional research is needed to understand how the internal school food environment can help support optimal diet quality in adolescents in the context of varying external food environments.

## Supplementary material

For supplementary material accompanying this paper visit https://doi.org/10.1017/S1368980022000994. click here to view supplementary material
