# Supplementary material for: Impact of the external school food environment on the associations of internal school food environment with high schoolers’ diet and BMI
Source: Public Health Nutr. 2022 Apr 25;25(11):3086–95. doi: 10.1017/S1368980022000994 (PMC9588460; doi:10.1017/S1368980022000994)
Supplement: Supplementary file 1 [file S1368980022000994sup.zip › S1368980022000994sup001.docx]

SUPPLEMENTARY TABLE 1: Sample and school characteristics in the NEXT full sample and the analytic subsample

| **Sample and school characteristics** | **NEXT full sample** | **Analytic subsample** | ***p* Value^1^** |
| --- | --- | --- | --- |
| **Students** | **n = 2,783** | **n = 2,263** |  |
| Age, years | 16.31 ± 0.56 | 16.30 ± 0.56 | 0.91 |
| Sex, female | 1,525 (54.86) | 1,242 (54.98) | 0.93 |
| BMI, kg/m^2^ | 24.70 ± 5.70 | 24.64 ± 5.64 | 0.72 |
| Race/Ethnicity, n (%) |  |  | <.0001 |
| White | 1,106 (39.93) | 1,014 (44.97) |  |
| Black | 687 (24.80) | 429 (19.02) |  |
| Hispanic | 835 (30.14) | 686 (30.42) |  |
| Other | 142 (5.13) | 126 (5.59) |  |
| Parent Education, n (%) |  |  | 0.61 |
| High school, GED, or less | 979 (38.71) | 832 (39.17) |  |
| Some college/tech school/Associates | 924 (36.54) | 782 (36.82) |  |
| Bachelor’s degree or higher | 626 (24.75) | 510 (24.01) |  |
| Family affluence, n (%) |  |  | 0.30 |
| Low affluence | 920 (33.06) | 719 (31.77) |  |
| Moderate affluence | 1,285 (46.17) | 1,056 (46.66) |  |
| High affluence | 578 (20.77) | 488 (21.56) |  |
|  |  |  |  |
| **Schools** | **n = 81** | **n = 72** |  |
| Neighborhood land use mix^2^ | 0.46 ± 0.30 | 0.45 ± 0.30 | 0.80 |
| Neighborhood population density (per square mile) | 7,674.8 ± 19,008.1 | 7,213.1 ± 19,818.1 | 0.88 |
| Neighborhood poverty rate^3^ | 36.05 ± 21.47 | 35.07 ± 21.29 | 0.78 |

Values are mean ± SD or n (%).

^1^ t-tests for continuous variables, Mann-Whitney U test for ordinal variables, and chi-square for categorical variables. Statistical significance at p = 0.05.

^2^ A score of 0-1 measured the diversity of land use in schools’ ZIP code tabulation area with 0 representing the most homogeneous land use and 1 representing the most diverse land use.

^3^ Percent of the population with income of less than 185% of the Federal Poverty Level.

SUPPLEMENTARY TABLE 2: Bivariate associations of student BMI and food intake frequency with demographic characteristics^1^

| Demographic characteristic | Student BMI (kg/m^2^) |  | FV intake (times/day) |  | Snacks intake  (≥1 time/day)^1^ |  | Soda intake  (≥1 time/day) |
| --- | --- | --- | --- | --- | --- | --- | --- |
|  | β (95% Cl) |  | β (95% Cl) |  | OR (95% CI) |  | OR (95% CI) |
| Race/Ethnicity |  |  |  |  |  |  |  |
| White | ref. |  | ref. |  | ref. |  | ref. |
| Black | 3.04 (2.23, 3.86)* |  | 0.97 (0.49, 1.44)* |  | 1.73 (1.32, 2.27)* |  | 1.75 (1.36, 2.24)* |
| Hispanic | 1.89 (1.15, 2.62)* |  | 0.47 (0.06, 0.89)* |  | 0.93 (0.71, 1.23) |  | 1.24 (0.99, 1.55) |
| Other | -0.90 (-2.18, 0.39) |  | 0.33 (-0.34, 1.00) |  | 1.03 (0.65, 1.62) |  | 0.72 (0.51, 1.05) |
| Parent Education |  |  |  |  |  |  |  |
| High school, GED, or less | ref. |  | ref. |  | ref. |  | ref. |
| Some college/tech school/Associates | -0.05 (-0.65, 0.55) |  | 0.56 (0.20, 0.91)* |  | 0.95 (0.77, 1.17) |  | 0.70 (0.57, 0.85)* |
| Bachelor’s degree or higher | -1.86 (-2.56, -1.16)* |  | 0.23 (-0.19, 0.64) |  | 0.84 (0.66, 1.08) |  | 0.47 (0.38, 0.59)* |
| Family affluence |  |  |  |  |  |  |  |
| Low affluence | ref. |  | ref. |  | ref. |  | ref. |
| Moderate affluence | -0.80 (-1.46, -0.14)* |  | -0.05 (-0.43, 0.32) |  | 0.92 (0.72, 1.17) |  | 0.94 (0.77, 1.14) |
| High affluence | -1.19 (-1.95, -0.42)* |  | -0.16 (-0.58, 0.27) |  | 1.01 (0.76, 1.33) |  | 1.00 (0.80, 1.25) |

^1^ Snack intake measured at wave 2; all other measures come from wave 1 (baseline).

Linear (FV intake) and logistic (snack and soda intake) mixed models were used to calculate estimates.

* p < 0.05


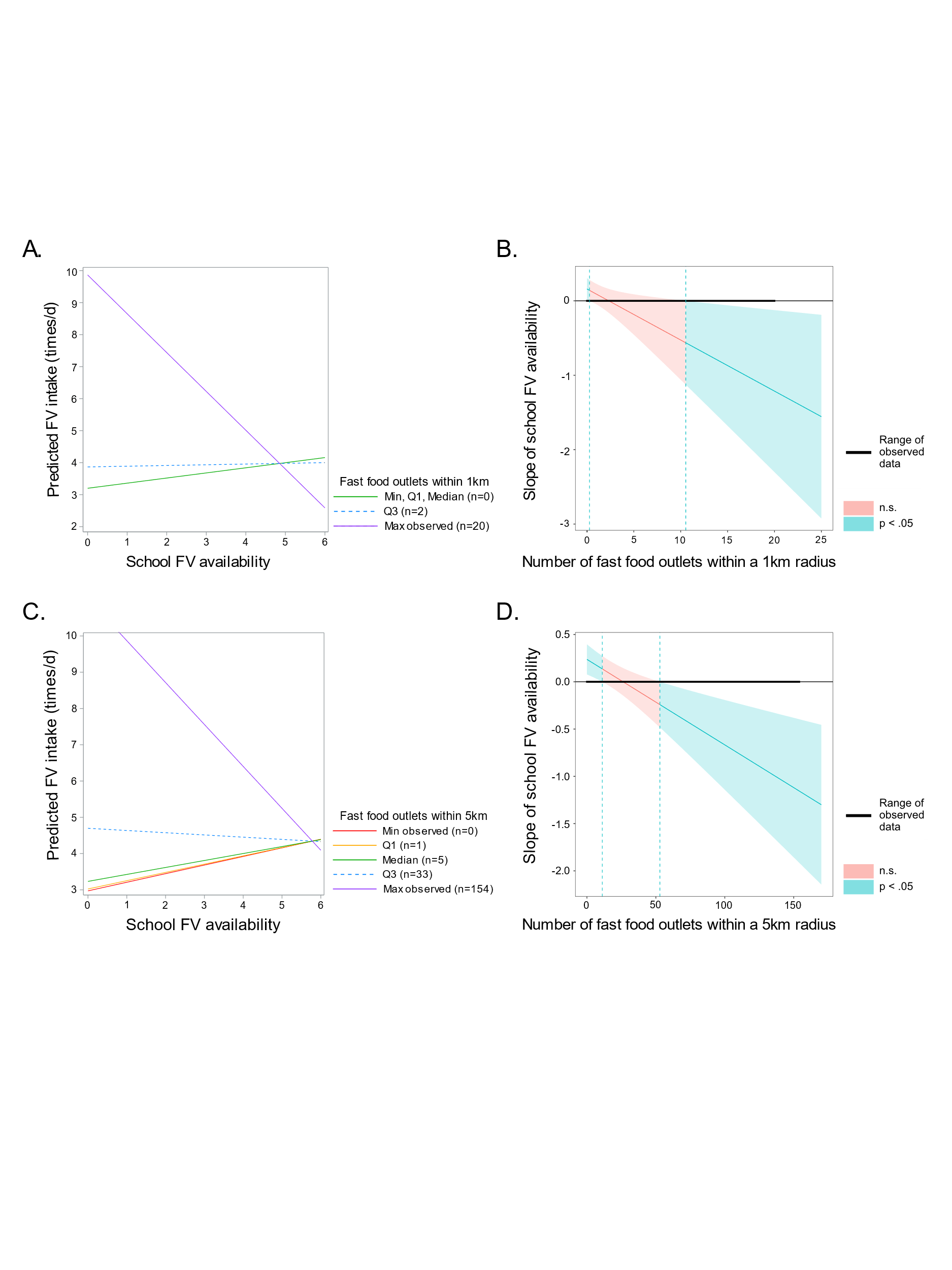


SUPPLEMENTARY FIGURE 1: **A.** Interaction plots showing simple slopes of the regression of school FV availability on student FV intake at different levels of fast food outlets within a 1 km radius of schools. Solid lines indicate that the slopes are within the regions of significance (p < 0.05) presented in Supplementary Figure 1B, while dashed lines indicate the slopes are outside of the regions of significance (p > 0.05) presented in Supplementary Figure 1B. **B.** Johnson-Neyman regions of significance and confidence bands (95% CI) for the conditional relation between school FV availability and student FV intake as a function of fast food outlets within 1 km of schools. Blue shaded areas reflect regions of significance ([0,0.27] and [>10.54]) and the bolden horizontal line indicates the range of observed food outlets in the sample data [0,20]. When the number of food outlets is 0 or above 10, the slope of school FV availability is p < 0.05. **C.** Interaction plots showing simple slopes of the regression of school FV availability on student FV intake at different levels of fast food outlets within a 5 km radius of schools. Solid lines indicate that the slopes are within the regions of significance (p < 0.05) presented in Supplementary Figure 1D, while dashed lines indicate the slopes are outside of the regions of significance (p > 0.05) presented in Supplementary Figure 1D. **D.** Johnson-Neyman regions of significance and confidence bands (95% CI) for the conditional relation between school FV availability and student FV intake as a function of fast food outlets within 5 km of schools. Blue shaded areas reflect regions of significance ([0,11.10] and [> 52.93]) and the bolden horizontal line indicates the range of observed food outlets in the sample data [0,154]. When the number of food outlets is between 0-11 and 53 and higher, the slope of school FV availability is p < 0.05.


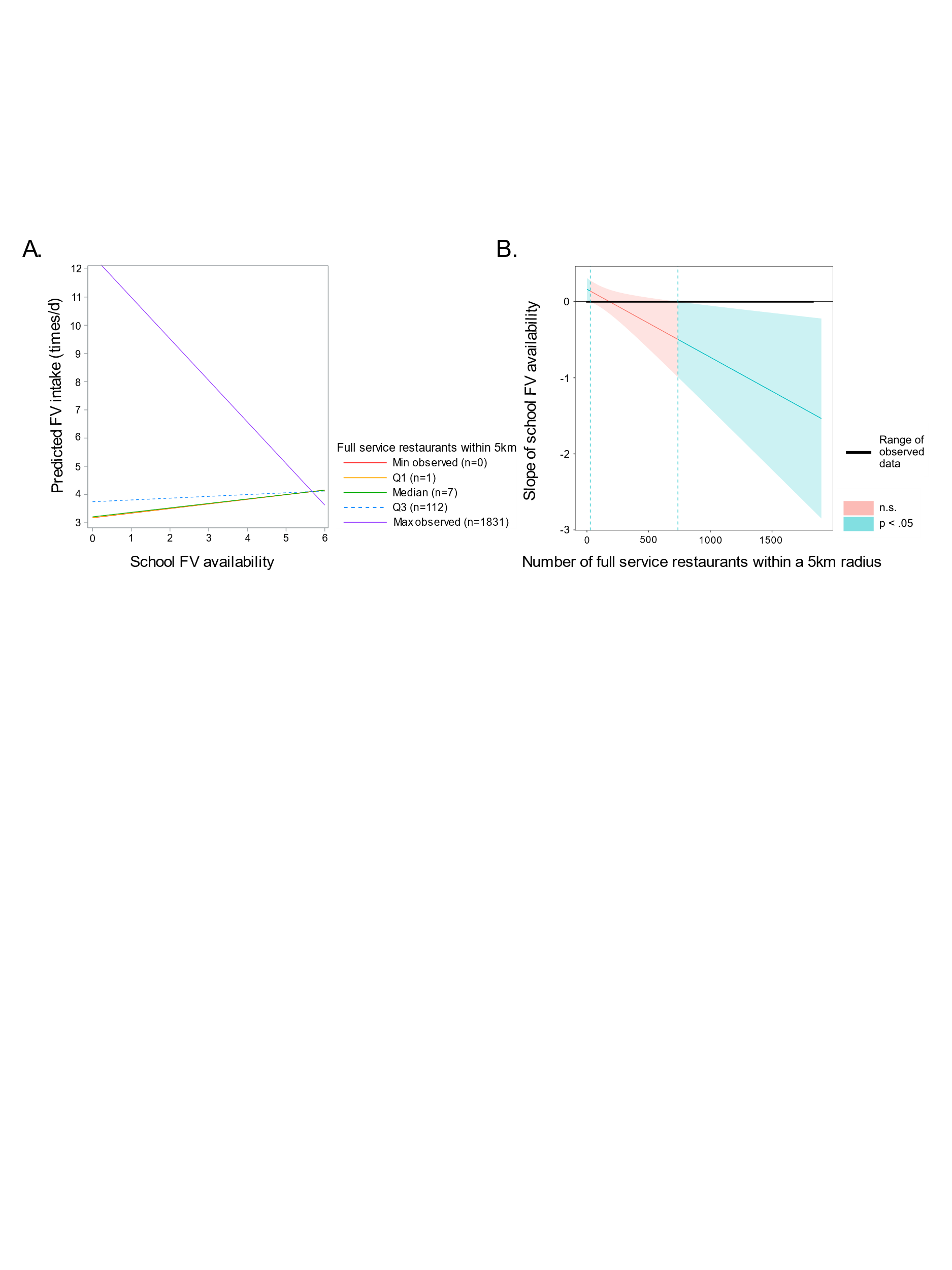


SUPPLEMENTARY FIGURE 2: **A.** Interaction plots showing simple slopes of the regression of school FV availability on student FV intake at different levels of full-service restaurants within a 5 km radius of schools. Solid lines indicate that the slopes are within the regions of significance (p < 0.05) presented in Supplementary Figure 2B, while dashed lines indicate the slopes are outside of the regions of significance (p > 0.05) presented in Supplementary Figure 2B. **B.** Johnson-Neyman regions of significance and confidence bands (95% CI) for the conditional relation between school FV availability and student FV intake as a function of full-service restaurants within 5 km of schools. Blue shaded areas reflect regions of significance ([0,26.3] and [> 737.6]) and the bolden horizontal line indicates the range of observed food outlets in the sample data [0,1831]. When the number of food outlets is between 0-26 and 738 and higher, the slope of school FV availability is p < 0.05.


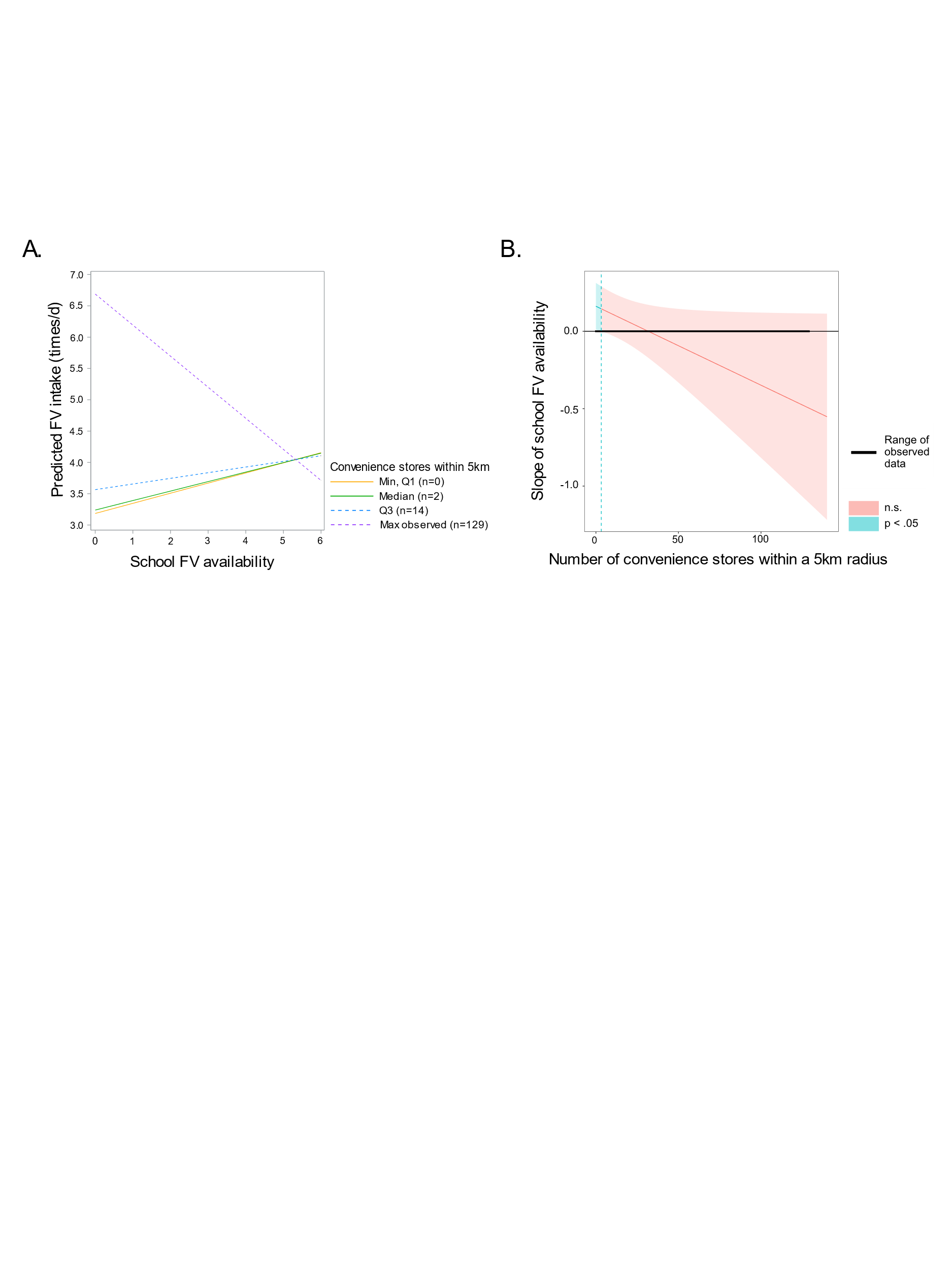
SUPPLEMENTARY FIGURE 3: **A.** Interaction plots showing simple slopes of the regression of school FV availability on student FV intake at different levels of convenience stores within a 5 km radius of schools. Solid lines indicate that the slopes are within the regions of significance (p < 0.05) presented in Supplementary Figure 3B, while dashed lines indicate the slopes are outside of the regions of significance (p > 0.05) presented in Supplementary Figure 3B. **B.** Johnson-Neyman regions of significance and confidence bands (95% CI) for the conditional relation between school FV availability and student FV intake as a function of convenience stores within 5 km of schools. Blue shaded areas reflect regions of significance [0,3.25] and the bolden horizontal line indicates the range of observed food outlets in the sample data [0,129]. When the number of food outlets is between 0 and 3, the slope of school FV availability is p < 0.05.
